# A method for measuring electrical signals in a primary cilium

**DOI:** 10.1186/2046-2530-1-17

**Published:** 2012-09-03

**Authors:** Nancy K Kleene, Steven J Kleene

**Affiliations:** 1 Department of Cancer and Cell Biology, University of Cincinnati, PO Box 670521, Cincinnati, OH, 45267–0521, USA

## Abstract

**Background:**

Most cells in the body possess a single primary cilium. These cilia are key transducers of sensory stimuli, and defects in cilia have been linked to several diseases. Evidence suggests that some transduction of sensory stimuli by the primary cilium depends on ion-conducting channels. However, the tiny size of the cilium has been a critical barrier to understanding its electrical properties. We report a novel method that allows sensitive, repeatable electrical recordings from primary cilia. Adherent cells were grown on small, spherical beads that could be easily moved within the recording chamber. In this configuration, an entire cilium could be pulled into a recording microelectrode.

**Results:**

In 47% of attempts, suction resulted in a seal with high input resistance. Single channels could be recorded while the cilium remained attached to the cell. When the pipette was raised into the air, the cell body was pulled off at the air-bath interface. The pipette retained the cilium and could then be immersed in various solutions that bathed the cytoplasmic face of the membrane. In excised cilia, ionic currents through ciliary channels were modulated by cytoplasmic Ca^2+^ and transmembrane voltage.

**Conclusions:**

Ciliary recording is a direct way to learn the effects of second messengers and voltage changes on ciliary transduction channels.

## Background

Most cells in the body possess a single primary cilium [1], a thin process that projects from the surface of the cell. Defects in primary cilia are implicated in a wide range of human pathologies [2-5], including cystic kidney disease [6]. Primary cilia are thought to be cellular antennae; they detect chemical, mechanical, osmotic, and gravitational stimuli [7-11]. Evidence indicates that some sensory stimuli may be transduced into electrical signals via ion channels in primary cilia (for examples, see [12,13]). Several channel proteins (TRPP2, TRPC1, TRPV4, α-epithelial sodium channel) have been localized to primary cilia [8,14-18]. Additional channels have been studied in specialized cilia from other cells (for example, sperm [19], *Chlamydomonas*[20], photoreceptors [21], and olfactory receptor neurons [22,23]) or found in the ciliary proteome [24-27].

Because of the primary cilium’s tiny size, direct examinations of the transduction of sensory to electrical signals in primary cilia are virtually non-existent. Instead, hypotheses of ciliary sensory function have been inferred from protein localizations, loss-of-function studies, and the effects of damaging or removing cilia [12,15,28]. There is now a substantial body of research characterizing the properties of ion channels relevant to primary cilia. Most investigators have taken indirect approaches, studying channel subunits expressed exogenously [17,29-33] or in artificial bilayers [13,16,30,34], or channels in non-ciliary cellular compartments [30,32,34]. The conclusions are sometimes noted to be contradictory [35-37], and it is difficult to guess how the channels might function in the native cilium. This limitation was acknowledged and addressed directly by Raychowdhury *et al.*[13,16]. Their investigations revealed channels in primary cilia isolated from cultured renal epithelial cells. To the best of our knowledge, their two reports are the only published recordings from the native membrane of any primary cilium.

Vision and olfaction also use specialized sensory cilia, and direct recordings from those cilia have long been abundant. It has been suggested that similar approaches might benefit research into primary cilia [4]. In 1991, we learned how to record from single olfactory cilia [38]. We have now adapted that method to allow sensitive, repeatable, and stable electrical recordings from single primary cilia, either cell-attached or immediately following detachment from a living cell. Our new method provides a direct means to investigate electrical signaling in the membrane of a primary cilium.

## Methods

### Cell culture

What follows is a description of the optimized culture and harvesting methods. mIMCD-3 cells (murine renal epithelial cells from the inner medullary collecting duct, CRL-2123, American Type Culture Collection, Manassas, VA USA [39]) were passaged weekly onto culture plastic with a 1:4 to 1:8 subcultivation ratio. The culture medium was Dulbecco’s modified Eagle’s medium (DMEM)/F12 (MT10092CV, Thermo Fisher Scientific, Waltham, MA USA) with 10% fetal bovine serum (SH30910.03, Thermo Fisher Scientific) and antibiotics (final concentrations 100 units/mL penicillin and 0.1 mg/mL streptomycin, SV30010, Thermo Fisher Scientific). Cells were incubated at 37°C in 95% air and 5% carbon dioxide.

**Figure 1:**
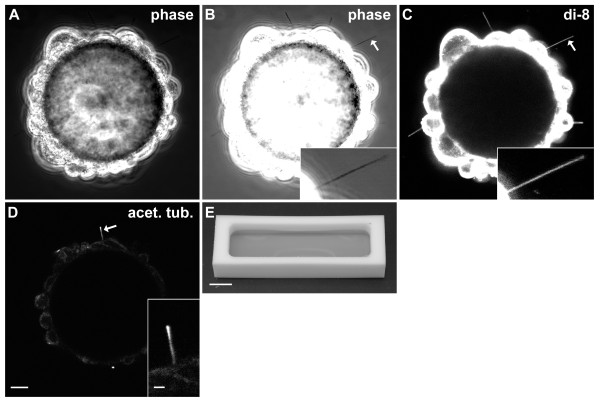
**Culture system for the growth of ciliated cells on beads.** (**A**-**B**) Phase-contrast image of live cells on a bead with contrast set to show cell bodies (**A**) or cilia (**B**). (**B** inset) Enlargement of cilium indicated by arrow. (**C**) Fluorescent image of bead from **A** and **B** labeled with di-8-ANEPPS (di-8). (**C** inset) Enlargement of cilium indicated by arrow. (**D**) Fluorescent image of a cell-coated bead fixed and immunolabeled for the ciliary marker acetylated α-tubulin (acet. tub.). (**D** inset) Enlargement of cilium indicated by arrow. (**E**) Teflon chamber with medium. Bar = 10 μm (**A**-**D**), 2 μm (**B**-**D** inset), 1 cm (**E**).

The glass-coated beads (G102- < 90, SoloHill Engineering, Ann Arbor, MI USA), were combined with distilled water (to 8 mg/mL) in a scintillation vial and autoclaved. For each milliliter of final chamber volume, 11.6 μL of the bead and water mixture was combined with 88.4 μL of medium in a sterile microcentrifuge tube and incubated at 37°C for at least 1 h. The autoclaved Teflon chambers were placed in a sterile Petri dish and incubated at 37°C for at least 1 h. Cells were dissociated with 0.05% trypsin/ethylenediaminetetraacetic acid (SH3023601, Thermo Fisher Scientific) for about 10 min. The dissociation was ended by the addition of medium with trituration. The cells were centrifuged at 300 × *g* for 5 min, liquid was removed, and the cells were resuspended in fresh medium with trituration. Cells (final, 2 × 10^5^ cells/mL), medium, and beads (final, 0.09 mg beads/mL, approximately 550 beads/mL) were placed in a depression that had been milled into a Teflon block (Figure1E, 8735K67, McMaster-Carr, Robbinsville, NJ USA). The external dimensions of the block were 76 mm × 26 mm × 13 mm; the dimensions of the depression were 65 mm × 17 mm × 11 mm. We used about 0.5 mL of cell suspension per square centimeter of Teflon chamber floor. The cells and beads were gently shaken for a few seconds every 15 min for the first 90 min of culture. We subsequently dispersed the beads with a cell lifter (70–2180, Biologix, Lenexa, KS USA); this significantly decreased the clumping of the beads. Cell-coated beads were usually used for recording after 10 to 15 days in culture. We used passages 17 to 31. Half of the growth medium was replaced every 2 to 3 days. To replace the medium, the chamber was held at an incline to give the beads time to settle before half of the medium was replaced. Chambers with a siliconized glass (Sigmacote, Sigma-Aldrich, St. Louis, MO USA) bottom were occasionally used, but we did not determine that they were better than Teflon chambers. However, they did permit monitoring of the culture more easily than the opaque Teflon, and at least some of the beads were free of the monolayer. In pilot studies, hollow borosilicate glass beads with a 10 μm diameter (AGSCO Corporation, Wheeling, IL USA) appeared to be engulfed by the cells and no cilia were visible.

On the day of use, half of the old medium was replaced with new. Then the Teflon chamber was held at an incline and the beads were allowed to settle before about 70% of the medium was removed. Next the chamber was tipped to gather beads near one end. Medium and beads were transferred to the well of a plastic culture plate (Nunc 176740, Thermo Fisher Scientific). The well had been pretreated with medium to reduce sticking. Eight rinses with sterile Ringer (see below for composition) were usually sufficient to remove debris and serum without damaging cilia or stripping the cells from the beads. Rinsing consisted of removal of all but about 270 μL of the fluid followed by the addition of 550 μL of Ringer. The Ringer was heated to 37°C and then allowed to cool to room temperature as aliquots were removed for rinsing. Rinsing beads in the plastic well allowed us to use the microscope to ensure that most of the beads returned to the bottom before removing fluid. We removed large clumps of cells or beads with tweezers. The beads and a minimal volume of Ringer were transferred to the recording chamber.

### Visualizing the cilia

To see the cilia during recording, we used a non-infinity-corrected, inverted microscope (Diaphot, Nikon, Tokyo, Japan) with a 40× Plan, phase-contrast objective (numerical aperture (NA) 0.7, DL, Ph3, Nikon), 15× ocular lenses (CFW, Nikon), and an extra-long-working-distance condenser (78924, NA 0.3, working distance 54 mm, Nikon). Phase-contrast microscopy cannot be used to visualize cilia lying above other cells or debris, so it was important that the bottom of the recording chamber (a cover glass) be clean. The cover glass was pretreated with filtered bovine serum albumin (10 mg/mL); this prevented the cell-coated beads from adhering to the glass [40]. A minority of the cilia had a small vesicular enlargement, usually at the distal tip (for example, cell 1 of Additional file 1).

As we learned to recognize the cilia and optimized the optics, it was helpful to use a fluorescent membrane dye to improve visibility. The dye, di-8-ANEPPS (D3167, Invitrogen / Life Technologies, Carlsbad, CA USA), was chosen because of its ability to label the membrane of live cells; we did not use its voltage-sensing properties. We mixed 1 μL of di-8-ANEPPS (1.7 mM stock in dry dimethyl sulfoxide) with 2.4 μL of 10% pluronic acid (Invitrogen / Life Technologies, P6866) before adding 497 μL of Ringer. Cell-coated beads were rinsed in Ringer, and 40 μL of the bead/Ringer mixture was added to the dye solution (final concentrations: 3 μM di-8-ANEPPS and 0.04% pluronic acid) for 20 min before rinsing again with Ringer. When dissolved in membrane, di-8-ANEPPS excites maximally at 465 nm and emits maximally at 635 nm. On the recording setup (described above), stained cilia were visualized with a fluorescein isothiocyanate (FITC) filter cube (DM510, Nikon) with a long-pass emission filter (BA515IF, Nikon). Cells used for electrical recording were never exposed to di-8-ANEPPS.

### Immunocytochemistry

Cell-coated beads were rinsed with DMEM/F12 and fixed for 15 min by adding 500 μL of 4% paraformaldehyde in 0.1 M phosphate buffer (30 mM KH_2_PO_4_, 0.1 M Na_2_HPO_4_, pH 7.2) to about 120 μL of beads and medium. The beads were rinsed with phosphate-buffered saline (PBS: 2.7 mM KCl, 1.5 mM KH_2_PO_4_, 8.1 mM Na_2_HPO_4_, 140 mM NaCl, pH 7.4) and blocked for 20 min with 2% normal rabbit serum, 0.1% Triton X-100, and 0.02% sodium azide in PBS. The monoclonal anti-acetylated α-tubulin antibody (mouse isotype IgG2b, ascites fluid, clone 6-11B-1, T6793, Sigma-Aldrich) had been raised against acetylated α-tubulin from the outer arm of *Strongylocentrotus purpuratus* (sea urchin). This antibody was diluted to 1/15,000 in antibody diluting buffer (0.2% Triton X-100 and 0.02% sodium azide in PBS). The antibody was applied for 1 h at room temperature. After rinsing with PBS, the fluorescently labeled rabbit anti-mouse antibody (Alexa 488, Jackson ImmunoResearch Laboratories, West Grove, PA USA, 1/500 in the antibody diluting buffer) was applied for 15 min. After rinsing in PBS, the beads were mounted with Fluoromount-G (SouthernBiotech, Birmingham, AL USA). The cilium in Figure1D is fairly straight and long, but cilia usually became shorter and less straight during fixation.

### Image acquisition and processing

**Figure 2:**
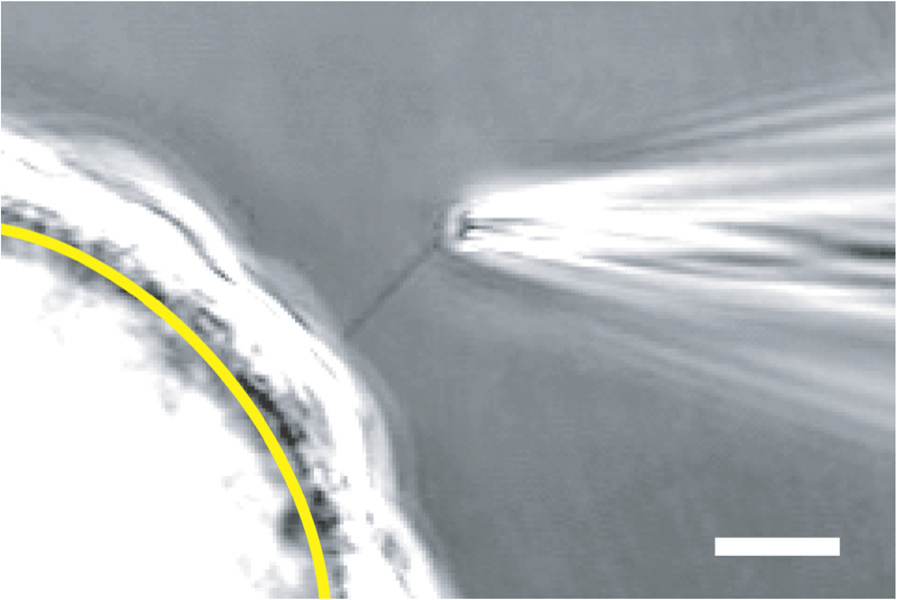
**Photomicrograph of the method for electrical recording from an isolated primary cilium.** A glass-coated bead is covered with a confluent layer of mIMCD-3 cells. The yellow line represents the boundary between the bead and the cells. One primary cilium of one cell is visible entering the tip of the recording pipette. Bar = 10 μm.

Images for Figure1A-D were acquired on an inverted, confocal microscope (LSM 710, Karl Zeiss AG, Oberkochen, Germany) and exported as TIFF files using Zen 2009 Light Edition (Zeiss). Figure1A-C were acquired with a 40×/0.75 NA, Plan-Neofluar, phase-contrast objective, 1400 pixels ×1400 pixels, 97 nm × 97 nm × 1,000 nm voxel dimensions, and 8-bit depth. The laser wavelength was 488 nm. For Figure1A,B, the transmission channel was used. For Figure1C, the dichroic mirror was set for 488 nm and the emission filter was 535 to 759 nm. Figure1D was acquired with a 63×/1.20 NA, C-Apochromat, water-immersion objective, 1,188 pixels × 1,188 pixels, 114 nm × 114 nm × 900 nm voxel dimensions, with 8-bit depth. The laser wavelength was 488 nm, the dichroic mirror was set for 488 nm, and the emission filter was 493 to 630 nm. Figure1E was acquired with a Nikon D70 single-lens reflex camera. For Figure1, Adobe Photoshop 6.0 was used for converting to grayscale, cropping, resizing, and adding labels. Photoshop was also used to adjust brightness and contrast, including gamma, via ‘Input Levels’. Figure1A-C were resampled from 97 to 114 nm/pixel X-Y resolution to match the resolution of Figure1D so that the same scale bar could be used for all four images. Inserts for Figure1B-D were resampled to match the greater resolution of the main figures. Figure1E was resampled to match the greater resolution of Figure1A-D. The videos in Additional file 1 were acquired with a camera (WV-CD50, Panasonic, Osaka, Japan), 1× TV relay lens (Nikon), and a digital video recorder (RDR-VX530, Sony, Tokyo, Japan). The videos were processed in mencoder (http://www.mplayerhq.hu) to increase gamma and contrast. Figure2 is the average of two sequential frames from Additional file 1, cell 2. Photoshop was used to crop and add the yellow arc and calibration bar to Figure2.

### Electrical recording

To record from intact single renal primary cilia, we modified a method developed for patch-clamping frog olfactory cilia [38,40]. Under phase-contrast microscopy at 600× magnification, a single primary cilium at least 10 μm in length was located. The tip of a recording micropipette was positioned near the distal tip of the cilium. Suction was applied through the pipette until the cilium began to enter the pipette. Entry of the cilium into the pipette was confirmed as follows. When half of the cilium had entered the pipette, the pipette was slowly moved back and forth in a direction perpendicular to its length. When the cilium had truly entered the pipette, it moved with the tip of the pipette, sometimes rotating the bead in the process ( Additional file 1). We have generally been unable to perform this test with cilia shorter than 10 μm. Additional suction was applied until the rest of the cilium entered the pipette and a high-resistance seal formed near the base of the cilium. The resistance sometimes increased over a period of several minutes, and suction up to −200 mmHg (−27 kPa) was used to make a seal. Only rarely did the membrane break to give a whole-cell recording. In most experiments, the pipette containing the cilium was then raised into the air and quickly re-immersed into a pseudointracellular solution. This served to excise the cilium from the cell, leaving the entire cilium inside the pipette in the inside-out recording configuration. In a few cases, this caused a loss of the high-resistance seal. Cilia were not studied further unless the input resistance (measured in the standard recording solutions described below) was at least 1 GΩ. The recording was often stable for at least 30 min, and it was routinely possible to transfer the pipette containing the excised cilium through the air to a series of baths containing different pseudointracellular solutions. A recording chamber built for this purpose has been previously described [40].

Recording micropipettes were pulled in two stages on a 700D vertical puller (David Kopf Instruments, Tujunga, CA USA) from capillaries of 8250 glass, a Schott borosilicate glass. Capillaries were fabricated by King Precision Glass, Inc. (Claremont, CA USA) to the dimensions outside diameter (OD) 1.5 mm, inside diameter (ID) 0.9 mm, and filament 0.1 mm. The best success was achieved when the pipette resistance was 7 to 10 MΩ in the standard recording solutions. High-resistance seals were also possible with blue-tipped microhematocrit capillaries of soda-lime glass (Thermo Fisher Scientific 22-362-574, ID 1.1 to 1.2 mm, wall 0.18 to 0.22 mm, no filament). However, the seals with the soda-lime glass were less stable, often breaking within two minutes.

During recording, the beads coated with cells were stored in a standard extracellular Ringer containing (in mM) NaCl 140, KCl 5, CaCl_2_ 2, MgCl_2_ 2, sodium pyruvate 2, 4-(2-hydroxyethyl)-1-piperazineethanesulfonic acid (HEPES) 5, and D-glucose 9.4, adjusted to pH 7.4 with NaOH. The recording pipettes also contained this solution. After excision of the cilium, the first (standard) pseudointracellular solution used to bathe the cytoplasmic face of the excised cilium contained (in mM) KCl 140, NaCl 5, CaCl_2_ 0.76, MgCl_2_ 2, HEPES 5, 1,2-bis(2-aminophenoxy)ethane-*N,N,N',N'*-tetraacetic acid (BAPTA) 2, and D-glucose 5, adjusted to pH 7.4 with KOH. The concentration of Ca_free_^2+^ in this solution was 0.1 μM. In other pseudointracellular solutions, Ca_free_^2+^ concentration was 3 μM or 300 μM. For the former, 1,2-bis(2-amino-5-bromophenoxy)ethane-*N,N,N',N'*-tetraacetic acid (dibromoBAPTA) was used instead of BAPTA.

All recordings were done under voltage-clamp at room temperature (24°C). The recording pipette and chamber were coupled by Ag/AgCl electrodes to an Axopatch 200B patch-clamp amplifier with a CV203BU headstage and Digidata 1200A BNC data-acquisition system, controlled by pCLAMP 5.7.1 software (all from Axon Instruments / Molecular Devices, Sunnyvale, CA USA). The pipette was positioned with an MO-103M hydraulic micromanipulator (Narishige, Tokyo, Japan). Currents were low-pass filtered at 2 kHz and digitized at 5 kHz. Software for analysis included Origin 7.0 (OriginLab Corporation, Northampton, MA USA) and QuB 1.5 (http://www.qub.buffalo.edu, State University of New York, Buffalo, NY USA). Results of repeated experiments are reported as mean ± standard error of the mean.

## Results

### Preparation of cells with primary cilia

To facilitate electrical recording, ciliated mIMCD-3 cells were grown on beads that were free to move within the recording chamber. This configuration allowed the full length of the cilium to follow suction applied through the recording micropipette until the pipette sealed near the base of the cilium. In culture, glass-coated beads (G102- < 90, diameter 63 ± 2 μm, *n* = 50, range 39 to 97 μm, SoloHill Engineering) became covered with ciliated cells (Figure1A). At the equator of the bead, a few cilia projected clearly beyond the cell layer and could be seen with phase-contrast microscopy (Figure1B). The diameter of a primary cilium is about 200 nm [41], near the limit of detection for a conventional light microscope. The fluorescent membrane dye di-8-ANEPPS (D3167, Invitrogen / Life Technologies) greatly enhanced the visibility of the live cilia (Figure1C). However, we did not use dye or fluorescent visualization when recording because of possible phototoxicity to the cilia [42]. We confirmed that the extensions were primary cilia by immunostaining for acetylated α-tubulin (Figure1D) with an antibody that is a well-recognized marker of primary cilia [43]. Due to their specific gravity (1.028), the beads sank to the bottom of the chamber; this facilitated changes of growth medium and rinsing. When the beads were cultured in a chamber with a glass bottom, the beads became embedded in a cellular monolayer that prevented them from being harvested easily. When the beads and cells were cultured in a Teflon chamber (Figure1E), many of the beads remained free of the monolayer.

### Electrical recording

**Figure 3:**
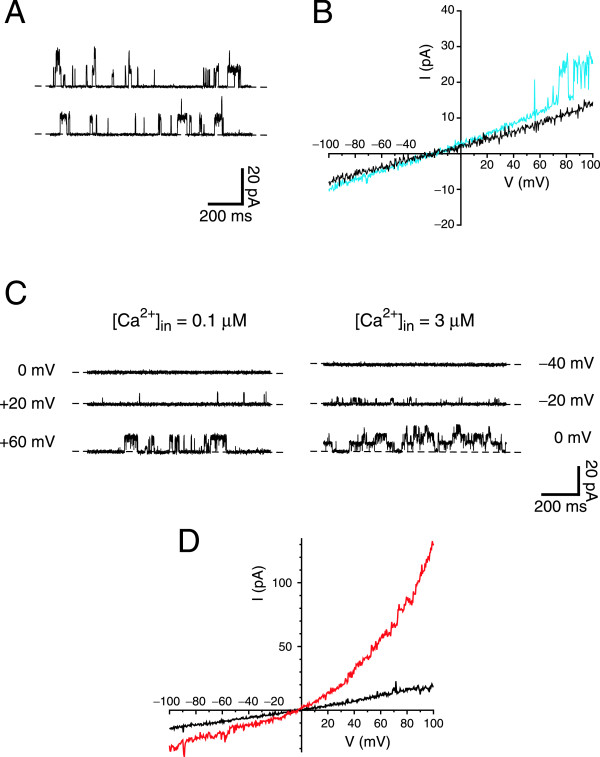
**Voltage-clamp recordings from single renal primary cilia.** All recordings were made in the standard recording solutions except as noted. In **A** and **C**, dashed lines indicate the current level when the channels were closed. (**A**) Single-channel fluctuations were recorded while the cilium in the pipette was still attached to the cell. Pipette potential was strongly depolarizing (−140 mV). (**B**) Membrane current–voltage relations of two cilia after excision from the cell. The recording shown in black has an input resistance R = 10.1 GΩ. In the recording shown in blue (R = 7.4 GΩ), single-channel openings are visible at potentials more positive than +50 mV. (**C**) Activation by Ca^2+^ and by depolarization of large-conductance channels in an excised renal primary cilium. On the left are shown the membrane currents at three voltage-clamp potentials in the presence of 0.1 μM free cytoplasmic Ca^2+^. On the right are recordings with 3 μM free cytoplasmic Ca^2+^ present. (**D**) A macroscopic current activated by high cytoplasmic Ca^2+^ in an excised cilium. The current–voltage relation was measured in each of two pseudointracellular baths: a bath containing 0.1 μM Ca^2+^ (black); and a bath with 300 μM Ca^2+^ (red).

Beads coated with ciliated cells were placed in a recording chamber and observed under phase-contrast microscopy. By applying positive pressure through the recording micropipette, it was often possible to rotate a bead until a cilium was positioned near the tip of the pipette. Suction was then applied through the pipette to draw the single cilium into the pipette (Figure2, Additional file 1). The cilium easily followed the suction because the bead was small and not attached to any surface. In 47% of 838 attempts, suction resulted in a membrane-pipette seal of resistance ≥  1 GΩ. With a cilium in the recording pipette and the cell still attached, bursts of single-channel activity were seen in a minority of cells, particularly at depolarizing potentials (Figure3A).

If the pipette was raised briefly into the air, the cell was pulled off at the air-bath interface, while the cilium remained sealed inside the pipette in the inside-out recording configuration. The pipette and cilium were then quickly transferred through the air and immersed in a solution that bathed the cytoplasmic face of the membrane. Such transfers could be repeated many times without rupturing the seal. Initially each cilium was placed in a pseudointracellular bath containing 0.1 μM free Ca^2+^. The current–voltage relation measured in this bath was linear (Figure3B), and the input resistance averaged 12.1 ± 0.4 GΩ (measured from −80 to +60 mV; *n* = 222, range 1.7 to 35 GΩ). Even in low Ca^2+^, some of the cilia showed single-channel fluctuations at strongly depolarizing potentials (Figure3B, blue; Figure3C). A preliminary study of this channel indicated that it had a conductance of 92 pS and a reversal potential of −65 mV in the standard recording solutions. It was activated by both depolarization and increasing cytoplasmic Ca^2+^ (Figure3C). This channel was seen in 32% of 38 cilia tested but was not detected in any of seven patches taken from the apical cell membrane.

In 85% of 47 cilia tested, 300 μM cytoplasmic free Ca^2+^ activated a second, distinct current (Figure3D). Clear single-channel events could not be discriminated, suggesting that the current reflects the summated activities of a moderate number of smaller-conductance channels. The Ca^2+^-dependent macroscopic current showed pronounced outward rectification and reversed near 0 mV in the standard recording solutions (Figure3D). A similar current was seen in 77% of 13 patches taken from the apical cell membrane.

## Discussion

Our goal was to allow the direct study of electrical signals in the membrane of a primary cilium. To develop a method for this, we selected the cilia of cultured renal epithelial cells for two reasons. First, as discussed below, an existing body of work has identified specific channels and receptors that are speculated to underlie sensory functions in renal cilia. Second, channel defects in renal cilia are implicated in cystic kidney diseases, including autosomal dominant polycystic kidney disease (ADPKD) [6], the most common monogenic renal disorder [44]. Most cases of ADPKD are caused by mutations in the genes [45] for either TRPP2, a channel protein located on the renal primary cilium, or polycystin-1, a ciliary molecule that interacts with TRPP2 [14,15,30,46,47].

In pilot studies, we were unable to record from the cilia of cells attached to fixed substrates such as cover glasses or folded filter paper [48]. On cover glasses, visual identification of the cilia was difficult because of the underlying cells. Even on folded filter paper, the base of the cilium could not be clearly seen. As a result, we were unable to move the recording pipette along the full length of a cilium to its base, where the pipette can seal to the membrane. This problem was overcome by growing the cells on a movable substrate, a glass-coated bead small enough to be easily moved by suction applied through the recording pipette. The full length of a cilium was able to follow this suction until the pipette sealed near the base of the cilium. Obtaining such seals was also possible with enzymatically dissociated cells, but that introduces a risk of enzymatic degradation of membrane proteins.

It is of central importance to know that the primary cilium enters the recording pipette. This was evident from direct observation (Figure2Additional file 1). The initial recording studies also suggested the presence of a specialized membrane. We identified a large-conductance channel (Figure3B,C) that was present in ciliary recordings but not in patches from the apical cell membrane. Although this method allows a focus on the ciliary membrane, it is likely that some plasma membrane surrounding the base of the cilium also enters the pipette [49]. Cytoplasmic effectors can be readily tested (for example, Ca^2+^, Figure3C,D), and it is possible to apply external stimuli via intrapipette perfusion [50]. Two limitations apply to our method at present. First, the cilium inside the pipette cannot be bent with fluid flow, which limits the ability to study mechanical stimulation. Second, the method used to verify that the cilium has entered the pipette (see Methods, Electrical Recording) is very difficult with cilia shorter than 10 μm. In the future, an appropriate ciliary marker might allow visualization of even a short cilium within the recording pipette following recording.

The first direct recordings from the membrane of a primary cilium were reported by Raychowdhury *et al.*[13,16]. Primary cilia were isolated from cultured cells derived from porcine renal epithelium, and membrane patches were excised from the cilia. Our method offers three improvements. First, we record from each cilium while it is cell-attached or immediately after it is plucked from the cell. In the previous method [16], isolating the cilia by centrifugation took over two hours. The cilia were then frozen and thawed for subsequent use. Second, recording from small excised patches [16] samples the membrane and reduces the chances of observing channels expressed at low density or at specific loci on the cilia. We record from the entire membrane of one cilium. Finally, the authors of the published method noted that low input resistances constrained their analyses [16]. As indicated above, we achieve very high input resistances.

The growth of ciliated cells on beads may prove useful in applications other than electrophysiological recording. Proteins have been isolated and identified from single cells, but the sensitivity is not high [51]. It is conceivable, particularly with longer cilia and future improvements in proteomic techniques, that micropipettes could be used to isolate enough cilia from cell-coated beads to generate a primary ciliome. We estimate that 420 cilia, each 40 μm long and 0.2 μm in diameter, would have a combined volume equal to that of a single cell 10 μm in diameter. An alternative approach would be to culture cells on beads in a spinner flask so that a large number of ciliated cells would be grown in a small space. Subsequent isolation of the cilia by established means [52] might suffice for the purpose of making a primary ciliome.

The method for recording from primary cilia was developed with cultured renal epithelial cells. We must acknowledge that cultured cells cannot perfectly represent the environment that exists in the inner medullary collecting duct of the murine kidney. There is evidence that protein localization to primary cilia is altered by environmental factors such as flow [53], availability of ligands [54], and serum deprivation [55]. However, culture conditions similar to ours have been successfully used by other researchers studying ciliary function in kidney cells [28,56]. In the future, it may be productive to use acutely dissociated preparations from patient samples despite the inherent risks of enzymatic damage to membrane proteins.

A technique that measures ciliary electrical activity will be useful in examining ciliary signaling pathways that use ion channels. Several channel proteins (TRPP2, TRPC1, TRPV4, α-epithelial sodium channel) and receptors (for example, epidermal growth factor receptor, type 2 vasopressin receptor) have been localized to renal primary cilia [13-18,57]. A wide variety of renal stimuli have been shown to alter the activity of these channels: TRPP2 (fluid flow [12], epidermal growth factor [57], pH [58], voltage [58], cytoplasmic calcium [31,34]); the TRPP2/TRPC1 complex (G-protein coupled receptor agonists [17]); and the TRPP2/TRPV4 complex (heat, swelling, external calcium [18]). It seems likely that different cells will have different channels localized to their primary cilia for the transduction of different stimuli. For example, TRPP2 is present on the primary cilium of the following cells and has been hypothesized or shown to transduce mechanical stimuli: embryonic node cells, ovarian granulosa cells, cholangiocytes, vascular smooth muscle cells, and vascular endothelial cells [59-63]. TRPV4 is present on the primary cilia of cholangiocytes and mediates a response to changes in osmolarity [8]. In neurons, several receptors for channel-containing pathways have been localized to primary cilia (for example, melanin-concentrating hormone receptor 1 [64,65]), although corresponding ciliary channels have not yet been found. The utility of our method should increase when coupled with methods for manipulating the expression of ciliary proteins. Targeted ciliary expression has been possible in IMCD [54,66] and other cell lines [67-69]. Recording from cells treated to knock down ciliary channel proteins should aid in identifying subunits contributing to channel function.

It would be helpful to show that our method confirms previous descriptions of ciliary membrane channels. Because such reports are rare, comparison is difficult. Raychowdhury *et al.* reported a channel resembling the epithelial sodium channel (ENaC) in cilia from the proximal tubule-like [70] cell line LLC-PK_1_[13]. Although ENaC has been found on the apical surface of cells from the inner medullary collecting duct [71], there are no reports of ENaC on the primary cilium in this portion of the nephron. We have not observed any constitutive Na^+^-selective current in the cilia of cells from the inner medullary collecting duct cell line mIMCD-3. The primary cilia of mIMCD-3 cells express the TRPC1 [17] and TRPP2 channel subunits [17,32]. In some systems, TRPP2 contributes to large-conductance channels that resemble the single channels we observed in mIMCD-3 cilia (Figure3A-C). However, the properties of TRPP2-dependent channel currents vary considerably depending on the cellular system and physiological solutions chosen [13,16-18,29-34,57,58]. Ultimately knockdown studies will be required to learn the molecular composition of the large-conductance channel in native mIMCD-3 cilia.

Methods for recording electrical events in the specialized cilia that underlie visual and olfactory sensation initiated over 20 years of rapid progress in both basic physiology and in the etiology of related human diseases (for example, retinitis pigmentosa and achromatopsia [21]). Our initial studies (Figure3) indicate that the ability to record from single primary cilia may allow similar advances. In cases where primary cilia underlie the transduction of a stimulus into an electrical signal, the method reported here should allow a direct means of investigation.

## Conclusions

We describe a novel method that allows for the first time electrical recording from a freshly isolated primary cilium with minimal contributions from other cellular compartments. Adherent cells were grown on small, spherical beads that could be easily moved within the recording chamber. In this configuration, an entire cilium could be pulled into a recording microelectrode. Many attempts yielded a seal with high input resistance and stability. Recording of ciliary electrical events was possible while attached to the cell and following excision of the cilium. Ciliary recording is a direct way to learn the effects of second messengers and voltage changes on ciliary transduction channels. It will enable resolution of the ambiguities that inevitably result from studies in non-native membranes. Ultimately, the culturing of ciliated cells on beads may also provide a means of gathering cilia for proteomics.

## Competing interests

The authors declare that they have no competing interests.

## Authors’ contributions

SJK and NKK designed the research and wrote the paper. NKK performed the cell culture, staining, and microscopy. SJK acquired electrophysiological data. Both authors read and approved the final manuscript.

## Supplementary Material

Additional file 1 **Video with six examples showing the method for electrical recording from an isolated primary cilium.** In each example, a glass-coated bead is covered with a confluent layer of mIMCD-3 cells. One primary cilium of one cell can be seen entering the tip of the recording pipette. Click here for file
